# UHPLC-PDA Assay for Simultaneous Determination of Vitamin D_3_ and Menaquinone-7 in Pharmaceutical Solid Dosage Formulation

**DOI:** 10.1155/2017/1208753

**Published:** 2017-08-21

**Authors:** Muhammad Jehangir, Mahmood Ahmed, Muhammad Imtiaz Shafiq, Abdul Samad, Iftikhar-ul-Haq

**Affiliations:** ^1^Department of Chemistry, Forman Christian College (A Chartered University), Lahore, Pakistan; ^2^NovaMed Pharmaceuticals, Lahore, Pakistan; ^3^Institute of Chemistry, University of the Punjab, Lahore 54590, Pakistan; ^4^Institute of Biochemistry and Biotechnology, University of the Punjab, Lahore 54590, Pakistan

## Abstract

A newly developed method based on ultrahigh performance liquid chromatography (UHPLC) was optimized for the simultaneous determination of vitamin D_3_ and menaquinone-7 (MK-7) in tablet formulation in the present study. UHPLC separation of vitamin D_3_ and MK-7 was performed with ACE Excel 2 C18-PFP column (2 *μ*m, 2.1 × 100 mm) at 0.6 mL min^−1^ flow rate, whereas the mobile phase consisted of methanol/water (19 : 1, v/v, phase A) and isopropyl alcohol (99.9%, phase B) containing 0.5% triethylamine. Isocratic separation of both the analytes was performed at 40°C by pumping the mobile phases A and B in the ratio of 50 : 50 (v/v, pH, 6.0). Both analytes were detected at a wavelength of 265 nm and the injection volume was 1.0 *μ*L. The overall runtime per sample was 4.5 min with retention time of 1.26 and 3.64 min for vitamin D_3_ and MK-7, respectively. The calibration curve was linear from 5.0 to 100 *μ*g mL^−1^ for vitamin D_3_ and MK-7 with a coefficient of determination (*R*^2^) ≥ 0.9981, while repeatability and reproducibility (expressed as relative standard deviation) were lower than 1.46 and 2.21%, respectively. The proposed HPLC method was demonstrated to be simple and rapid for the determination of vitamin D_3_ and MK-7 in tablets.

## 1. Introduction

**Figure 1:**
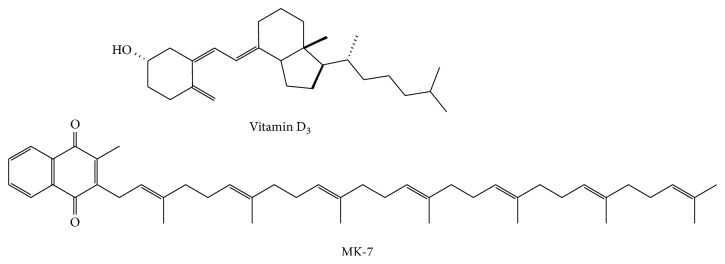
Molecular structures.

Vitamin D_3_ (cholecalciferol) is chemically known as (3*β*,5Z,7E)-9,10-secocholesta-5,7,10(19)-trien-3-ol (C_27_H_44_O, 384.64 g/mol) which belongs to fat soluble secosteroids group. Vitamin D_3_ can be ingested from the diet and supplements but it is naturally produced by human body after UVB (280–315 nm) radiation exposure. Active form of vitamin D_3_ (1*α*, 25 (OH)_2_ D_3_) exists in liver and kidney after hydroxylation [1, 2]. Vitamin K belongs to class of fat soluble vitamins comprising a number of structurally related compounds including vitamin K1 (phylloquinone) and vitamin K2s (menaquinones). Menaquinone-7 (MK-7) is the most important nutrition and all-trans menaquinone-7 is the active form. MK-7 is chemically known as 2-[(2E,6E,10E,14E,18E,22E)-3,7,11,15,19,23,27-heptamethyloctacosa-2,6,10,14,18,22,26-heptaenyl]-3-methylnaphthalene-1,4-dione (C_46_H_64_O_2_, 649.02 g/mol). The chemical structures for both components are depicted in Figure 1. Menaquinones are produced by bacteria in process of fermentation; however, it can be produced synthetically [3, 4]. Imbalance diet and acute or chronic illness are most frequently induced nutritional deficiency. Nutritional deficiency can also be provoked by medical treatment and surgical stress. Nutritional deficiencies, particularly vitamins imbalance, are caused by pharmacological agents like folate antagonists, anticoagulants, diuretics, antacids, oral hypoglycemic agents, antibiotics, anesthetic agents, and psychotropic agents [5]. Nonabsorbed sucrose polyester and drugs that induced changes in lipid processing in the gastrointestinal tract are responsible for deficiencies of lipid soluble vitamins (vitamin D_3_ and MK-7). Vitamins D_3_ and K deficiencies occur due to induction of Cyp450 and eradication of bacteria (responsible for synthesis of vitamin K), respectively [6–8]. Many scientific papers described the analysis of vitamin D_3_ and MK-7 in various matrixes such as biological fluids, foods, plant material by fluorimetric, UV-Vis, and MS detection after chromatographic separation [9–14]. Determination of vitamin K is also reported by HPLC coupled to chemiluminescence and electrochemical detectors [15, 16]. Using the above-mentioned techniques, a good sensitivity can be achieved but cost and complexity associated with these methods are problematic for routine analysis in quality control (QC) laboratories of pharmaceutical industries. So we need to develop a rapid and cost effective method for routine analysis of vitamin D_3_ and MK-7 in QC laboratories. The proposed method describes the optimization of UHPLC method and its comparison with HPLC. HPLC is prominent technique in laboratories for the last 30 years but did it not keep the pace with growing demand of analysis in short interval of time with reliability. Using UHPLC, more samples can be analysed in very short time with reliability and accuracy. Eddy and longitudinal diffusion coefficients in Van Deemter equation play important role regarding the separation of analytes. These coefficients are directly related to particle size of column packing and decreased with small particle size which results in better resolution [17–20]. The goal of present study was to optimize and validate the UHPLC method for simultaneous determination of vitamin D_3_ and MK-7 in tablet formulations. The developed method was compared with HPLC to prove its adequacy for pharmaceutical studies with minimum consumption of solvents, high resolution, and symmetrical peaks. ICH (International Council for Harmonization) guidelines [21–24] were followed to validate the proposed UHPLC method.

## 2. Experimental

### 2.1. Chemicals and Reagents

Vitamin D_3_ and MK-7 reference standard were provided by Sichuan Kelun Pharmaceutical Co., Ltd., China, and Gnosis Bioresearch SA, Switzerland. Acetic acid, glacial (AcOH), methanol (MeOH), ethanol (EtOH), triethylamine (TEA), and isopropyl alcohol (IPA) were supplied by Honeywell (USA). All the chemicals and reagents were of analytical grade, while GenPure water system (Thermo Scientific, USA) was used to obtain ultrapure water (18 MΩ·cm^−1^).

### 2.2. Chromatography

For HPLC analysis, Shimadzu Japan, liquid chromatographic system (LC-20A), with diode array detector (SPDM20A) and online degasser (DGU-20A5) equipped with ACE 5 C18 column (5 *μ*m, 4.6 × 250 mm), was used. For UHPLC analysis, Shimadzu Japan, liquid chromatographic system (Naxera 2, LC-30AD), with diode array detector (*i*-DReC, SPD-M30A) and online degasser (DGU-20A5) equipped with ACE Excel 2 C18-PFP column (2 *μ*m, 2.1 × 100 mm), was used. Both the systems were equipped with autosampler (SIL-20AXR) with injection volume ranging between 0.1 and 50 *μ*L. Mobile phase A is comprised of MeOH/H_2_O (19 : 1), while mobile phase B was IPA (HPLC grade, 99.9%) and both were pumped in ratio of 50 : 50 (v/v) at pH 6.0 adjusted by AcOH (pH meter, Orion 5 Star, Thermo Scientific, UK), whereas 0.5% (v/v) was used as silanol blocker. 5.0 *μ*L and 1.0 *μ*L injection volume were injected and flow rate was set at 1.0 and 0.6 mL min^−1^ for HPLC and UHPLC, respectively. The detection was carried out at 25°C and 40°C, respectively, for HPLC and UHPLC with best selected wavelength of 265 nm by *i*-DReC (detector). Shimadzu LC program (Lab Solutions Software) was used to record chromatograms, peak quantification, and integration. Mobile phase, standard solutions, and samples were filtered through nylon filter (0.45 *μ*m, Sartorius, Germany) before injection into chromatographic system.

### 2.3. Standard and Working Solutions

Individual stock standard solution of vitamin D_3_ and MK-7 (1000 *μ*g mL^−1^) was prepared in ethanol in ultrasonic bath for 15 min and working solutions of vitamin D_3_ and MK-7 were prepared from stock standard solution in mobile phase. Mixed standard solutions of vitamin D_3_ and MK-7 (25.0 *μ*g mL^−1^ each) were also prepared by diluting the stock standard solution in mobile phase.

### 2.4. Analysis of Tablet Formulation by Standard Addition

The stated composition of tablet (Avelia®) is vitamin D3 (10 *μ*g) and MK-7 (90 *μ*g) was analysed by the proposed method. Twenty tablets were grinded and aliquots equivalent to one tablet were diluted with ethanol containing 1.24 mg of vitamin D_3_ and 1.16 mg of MK-7 in 50 mL flask and final concentration of each analyte became 25 *μ*g mL^−1^. Then they were sonicated for 15 min for complete dissolution and finally diluted with mobile phase.

### 2.5. Validation Studies

Validation studies were performed to characterize the proposed analytical method such as specificity, linearity, accuracy, precision, limit of detection (LOD), limit of quantitation (LOQ), and conformity of chromatographic parameters (tailing factor, selectivity factor, resolution, and theoretical plates). Conformity of chromatographic conditions is basically system suitability tests which are foremost part of validation studies. So system suitability tests were performed in a prior step of validation studies.

### 2.6. Specificity

Analysis of placebo was performed to assess the specificity of the proposed chromatographic method [24, 25]. Sodium starch glycolate, magnesium stearate, sodium lauryl sulphate, polyvinyl povidone (PVP-K30), and polyethylene glycol (PEG-6000) were dissolved in ethanol and dilutions were made in mobile phase for specificity studies.

### 2.7. Linear Dynamic Range and Linearity

For both LC-based methods (HPLC and UHPLC), the linear dynamic range was selected within 5.0–100 *μ*g mL^–1^ for both analytes. A linear calibration curve in the form of *y* = *ax* + *b* was obtained by plotting the peak area *y* against the nominal concentration *x* of seven concentrations (5.0, 10.0, 15.0, 25, 50.0, 75.0, and 100 *μ*g mL^−1^), whereas a represented slope of the calibration curve and *b* indicated the intercept. Linear regression equation was demonstrated and tabulated the necessary parameters.

### 2.8. Accuracy and Precision

The accuracy of each method was determined in triplicate by spiking a known amount of each analyte standard solution in the dosage form (10 *μ*g tablet content + 1240 *μ*g standard added in 50 mL = 25 *μ*g mL^−1^ vitamin D_3_ and 90 *μ*g tablet content + 1160 *μ*g standard added in 50 mL = 25 *μ*g mL^−1^) resulting in final concentrations of 37.5, 50.0, and 62.5 *μ*g mL^−1^, for vitamin D_3_ and MK-7. This represented 50, 100, and 150% of each analyte in the dosing formulation. For precision determination, vitamin D_3_ and MK-7 were spiked at 20.0, 25.00, and 30.0 *μ*g mL^–1^ representing 80, 100, and 120% of each analyte, resulting in final concentrations of 45.0, 50.0, and 55.0 *μ*g mL^−1^, for each analyte. The intraday precision (repeatability) was evaluated by replicates of five on one day, whereas the interday precision (reproducibility) was determined over three consecutive days.

### 2.9. Method's LOD/LOQ

Vitamin D_3_ and MK-7 standard solution was injected in replicates of six. The resultant parameters of the linear regression including the standard deviation (SD) of the response based upon the slope *a* and intercept *b* determined the LOD and LOQ of the UHPLC method. The LOD and LOQ were defined as 3.3 *σ*/*S* and 10 *σ*/*S*, respectively [26–29], where *σ* is standard deviation and *S* is slope of regression line.

### 2.10. Method Robustness

Small but deliberate changes in chromatographic conditions such as mobile phase, pH, column temperature, and flow rate were done to evaluate the robustness of the proposed UHPLC method.

## 3. Results and Discussion

### 3.1. Optimization of Chromatographic Conditions

**Figure 2:**
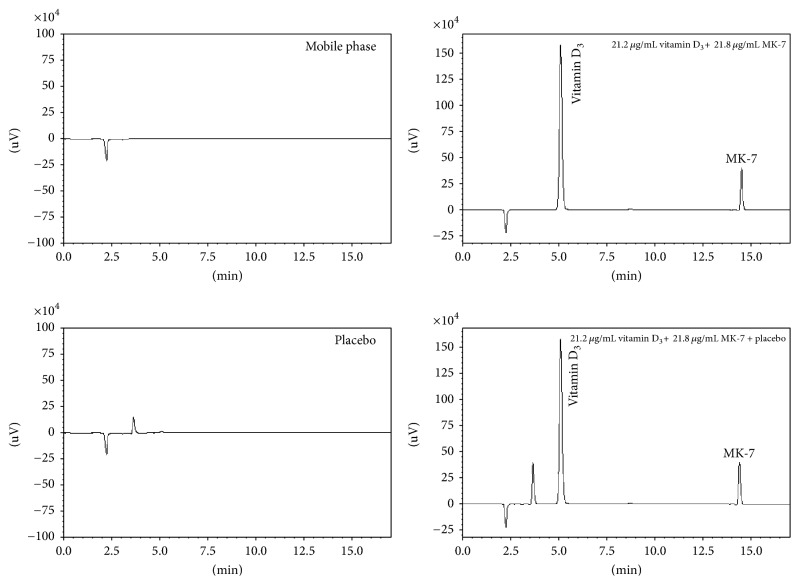
Typical HPLC chromatograms of vitamin D_3_ and MK-7 with and without placebo.

**Figure 3:**
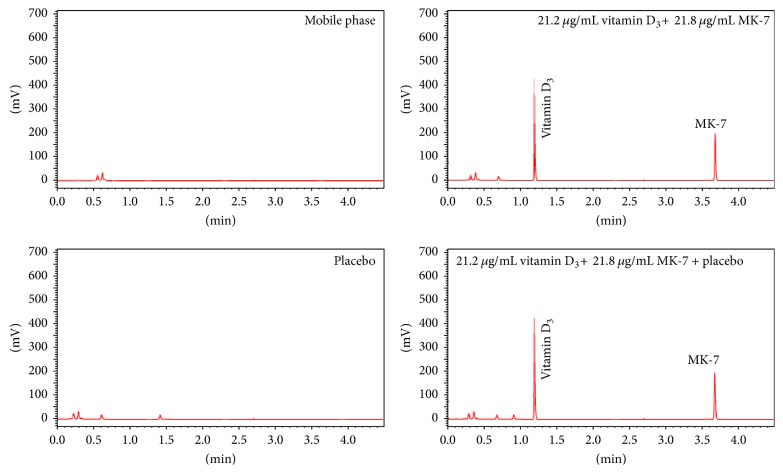
Typical UHPLC chromatograms of vitamin D_3_ and MK-7 with and without placebo.

Optimization of HPLC and UHPLC method was done by performing system suitability tests; in range of 200–400 nm, both vitamin D_3_ and MK-7 were scanned and absorption spectrum was noted. Both analytes were absorbed in this range with absorption maximum at 265 nm. Four different mobile phase (*A* : *B*) compositions such as 60 : 40, 50 : 50, 40 : 60, and 30 : 70 were examined to optimize chromatographic conditions such as tailing factor (*T* ≤ 2), selectivity factor (*α* > 1), resolution (Rs > 2), and theoretical plates (*N* > 2000) to get compliance with ICH guidelines (Table 1). For HPLC, these mobile phases were run on different columns like ACE 5 C18, Venusil XBP C18, Hypersil ODS, and Purespher® RP-18, while ACE Excel 2 C18-PFP, Waters ACQUITY 1.7 BEH C 18, Agilent Poroshell 2.7 120 EC C18, and Phenomenex Kinetex 2.6 C18 were employed with UHPLC at different pH (3.0, 4.0, 5.0, and 6.0). The typical chromatograms of vitamin D_3_ and MK-7 with and without placebo obtained by both the LC-based methods are presented in Figures 2 and 3. Free silanol in column packing could be interacting with drugs of both acidic and basic nature. In order to improve the peak shapes, TEA, a silanol blocker, was added to the mobile phase (0.5%, v/v). Silanol blocker provided additional selectivity by *π*-*π* and dipole interaction which resulted in achieving the better overall resolution [30].

In the end, the mobile phase consisting of MeOH/H_2_O and IPA in a ratio of 50 : 50 (v/v) with the addition of 0.5% TEA was found to be excellent using ACE 5 C18 and ACE Excel 2 C18-PFP columns for UHPLC and HPLC analysis, respectively. The chromatographic parameters under final conditions are summarized in Table 2 exhibiting an excellent peak shape, resolution, and higher number of theoretical plates.

### 3.2. Validation Studies

The specificity of the optimized UHPLC method was examined with vitamin D_3_ and MK-7 at concentration each of 25.00 *μ*g mL^–1^, relative to the blank mobile phase (Figure 3). The presence of placebo did not interfere during the determination of vitamin D_3_ and MK-7 as the components were baseline separated. For both chromatographic methods over a dynamic range of 5.0–100 *μ*g mL^–1^, seven concentrations (5.0, 10.0, 15.0, 25, 50.0, 75.0, and 100 *μ*g mL^−1^) were employed to construct a calibration graph for vitamin D_3_ and MK-7. The calibration curves were linear for vitamin D_3_ and MK-7 with a coefficient of determination (*R*^2^) ≥ 0.9981 regardless of the LC-based method (Table 3). Accuracy of methods by both the techniques under investigation was performed by evaluating the recovery studies after spiking the known amount of standard drugs in commercial products.

LOD and LOQ determined by UHPLC were 1.5-fold less than HPLC which is due to much sensitive detector *i*-DReC employed in UHPLC. The recovery results were obtained between the ranges of 98.97–101.74% and 99.36–101.56% (Table 4) for HPLC and UHPLC, respectively, which justified the suitability of the techniques for their intended applications. In addition, the results obtained were not differing significantly among the tested methods (HPLC and UHPLC) employed for determination of vitamin D_3_ and MK-7 at 95% of confidence interval. All the experimental *t*-values and *F*-values (Table 4) were below the theoretical *t*-values (4.30) and *F*-values (19.0). Advantages of UHPLC over HPLC were its rapidity, ease of operation, high selectivity, and consumption of minimum amount of solvents.

For precision studies, the results of repeatability and reproducibility are presented in Table 5 by injecting three different concentrations (80, 100, and 120% level of analyte under investigation) of standard solutions of vitamin D_3_ and MK-7 (*n* = 5) on the same day and three consecutive days, respectively. RSD values for repeatability and reproducibility were obtained less than 1.92 and 2.30, respectively, for HPLC and less than 1.46 for repeatability and 2.21 for reproducibility assays with UHPLC.

### 3.3. Method Robustness

The robustness of the proposed UHPLC method was evaluated by slight changes of the chromatographic parameters including the flow rate (±0.1 mL min^−1^), mobile phase ratio (±5.0 mL), column temperature (±5°C), wavelength (±2 nm), and pH (±0.1). Afterwards, the drug contents besides chromatographic parameters like retention time, tailing factor, number of theoretical plates, and resolution were determined. The results summarized in Table 6 demonstrated that the effects of the deliberate changes in chromatographic conditions are neglectable and that the proposed UHPLC method was robust for its intended applications.

### 3.4. Analysis of Commercial Tablet Formulation

The applicability of the proposed UHPLC method was evaluated by examining the commercial tablet (Avelia) with reported concentration of vitamin D_3_ (10 *μ*g) and MK-7 (90 *μ*g). Since the tablet contained the microcontents of both the analytes, for better analysis performance and to get reliable assay results, standard addition method was adopted. It was ensured that the removal of the excipients with an extraction step before analysis was unnecessary. It was concluded that the proposed UHPLC method was sufficiently accurate and precise (Table 7) with recovery and RSD found was 103.59, 102.87% and 1.20, 1.12% for vitamin D_3_ (10 *μ*g) and MK-7 (90 *μ*g), respectively.

## 4. Conclusion

In the literature, UHPLC method for simultaneous determination of vitamin D_3_ and MK-7 in pharmaceutical formulations is not found available. For this reason, UHPLC method was fully validated according to ICH guidelines and was presented for determination of vitamin D_3_ and MK-7 in tablet formulations. Remarkable advantages of UHPLC over HPLC were found such as rapidity, ease of operation, high selectivity, and consuming minimum amount of solvents. Good recoveries, interference-free, and high reproducible chromatograms were achieved. The proposed method was optimized step by step and presented its suitability for quality control laboratories where time and economy are essentially required. The proposed method showed its adequacy with high recovery in the presence of excipients and additives used in the formulations.

## Figures and Tables

**Table 1:** Results of tested stationary phase.

Column	Analyte	*R*_*s*_	*T*_*f*_	*α*	*N*
HPLC					
Hypersil ODS (250 × 4.6 mm, 5 *μ*m)	Vitamin D_3_	—	1.11	—	4132
	MK-7	13.14	1.36	5.64	8329
Venusil XBP C18 (250 × 4.6 mm, 5 *μ*m)	Vitamin D_3_	—	1.14	—	3763
	MK-7	12.54	1.31	5.31	7296
ACE 5 C18 (250 × 4.6 mm, 5 *μ*m)	Vitamin D_3_	—	1.11	—	5867
	MK-7	15.92	1.12	6.83	9683
Purespher® RP-18 (250 × 4.6 mm, 5 *μ*m)	Vitamin D_3_	—	1.37	—	3923
	MK-7	13.45	1.09	5.91	8209

UHPLC					
ACE Excel 2 C18-PFP (100 × 2.1 mm, 2 *μ*m)	Vitamin D_3_	—	0.94	—	23289
	MK-7	27.61	0.98	4.61	28521
Waters ACQUITY 1.7 BEH C 18 (100 × 2.1 mm, 2 *μ*m)	Vitamin D_3_	—	1.32	—	19342
	MK-7	24.23	1.15	4.25	25029
Agilent Poroshell 2.7 120 EC C18 (100 × 2.1 mm, 2 *μ*m)	Vitamin D_3_	—	1.23	—	15687
	MK-7	21.54	1.15	3.71	22143
Phenomenex Kinetex 2.6 C18 (100 × 2.1 mm, 2 *μ*m)	Vitamin D_3_	—	1.07	—	11981
	MK-7	19.21	1.12	3.31	19814

**Table 2:** System suitability test parameters.

Parameters	Analyte/technique			
	HPLC		UHPLC	
	Vitamin D_3_	MK-7	Vitamin D_3_	MK-7
Retention time (*t*_*R*_ in min)	5.11	14.49	1.26	3.64
Tailing factor (*T*)	1.11	1.12	0.94	0.98
Resolution (Rs)	—	15.92	—	27.61
Selectivity factor (*α*)	—	6.83	—	4.61
Theoretical plates (*N*)	5867	9684	23289	28521
% RSD of retention time (*t*_*R*_)	0.003	0.002	0.002	0.004

**Table 3:** Statistical evaluation of regression data of vitamin D_3_ and MK-7 by HPLC and UHPLC.

Parameters	Analyte/technique			
	HPLC		UHPLC	
	Vitamin D_3_	MK-7	Vitamin D_3_	MK-7
Linearity range (*μ*g mL^−1^)	5–100	5–100	5–100	5–100
Slope	1998606	136363	0.5854	0.5854
Intercept	5725724	70561.4	134.52	104.52
Standard error of slope	92902	6808	3.6 × 10^−2^	4.43 × 10^−2^
Standard error of intercept	469133	34377	1.181	1.901
Coefficient of determination (*R*^2^)	0.9984	0.9981	0.9984	0.9981
Limit of detection (*μ*g mL^−1^)	0.24	0.27	0.16	0.18
Limit of quantification (*μ*g mL^−1^)	0.72	0.81	0.48	0.54

**Table 4:** Accuracy studies of vitamin D_3_ and MK-7 by HPLC and UHPLC.

Analyte	^a^Concentration after spiking (*μ*g mL^−1^)	HPLC		UHPLC		^c^ *t*-experimental [*F*-experimental]
		^b^Concentration found (*μ*g mL^−1^) ± SEM; RSD	(%) recovery [BIAS]	^b^Concentration found (*μ*g mL^−1^) ± SEM; RSD	(%) recovery [BIAS]	
Vitamin D_3_	37.5	37.53 ± 0.21; 1.18	100.08	37.55 ± 0.22; 1.48	100.13	0.64 [4.4]
			[0.08]		[0.13]	
	50.0	50.87 ± 0.18; 0.71	101.74	50.78 ± 0.21; 0.71	101.56	−4.16 [8.7]
			[1.74]		[1.56]	
	62.5	62.48 ± 0.17; 0.43	99.97	62.57 ± 0.14; 0.33	100.11	−1.15 [0.79]
			[0.03]		[0.11]	

MK-7	37.5	37.12 ± 0.22; 2.08	98.97	37.26 ± 0.32; 2.55	99.36	0.52 [0.55]
			[1.01]		[0.64]	
	50.0	50.56 ± 0.21; 0.98	101.12	50.58 ± 0.42; 1.16	101.16	−0.29 [0.46]
			[1.12]		[1.16]	
	62.5	62.79 ± 0.24; 1.98	100.46	62.27 ± 0.51; 0.40	99.63	5.77 [0.32]
			[0.46]		[0.83]	

^a^Actual concentration of vitamin D_3_ and MK-7 = 25 *μ*g mL^−1^. ^b^All measurements were made in triplicate; SEM: standard error mean; RSD: relative standard deviation. ^c^Theoretical *t*-value is 4.30 and *F*-value is 19.0, at *p* = 0.05.

**Table 5:** Precision studies of vitamin D_3_ and MK-7 by HPLC and UHPLC.

Analyte	Repeatability (*n* = 5)		Reproducibility (*n* = 5)		
	Concentration (*μ*g mL^−1^)	Concentration found (*μ*g mL^−1^) ± SEM; RSD	Concentration found (*μ*g mL^−1^) ± SEM; RSD		
			Day 1	Day 2	Day 3
Technique: HPLC					
Vitamin D_3_	45.0	44.94 ± 0.23; 1.45	44.92 ± 0.81; 0.61	44.93 ± 0.52; 1.41	45.21 ± 0.42; 1.03
	50.0	50.41 ± 0.11; 0.42	49.95 ± 1.22; 0.84	50.65 ± 0.42; 1.00	50.42 ± 0.73; 1.19
	55.0	54.98 ± 0.36; 1.70	55.49 ± 0.73; 1.20	55.43 ± 0.32; 0.80	55.44 ± 0.43; 1.01
MK-7	45.0	44.92 ± 0.22; 1.92	44.98 ± 0.32; 2.30	45.24 ± 0.23; 2.23	45.24 ± 0.12; 1.98
	50.0	49.46 ± 0.31; 1.02	50.58 ± 0.12; 1.72	50.25 ± 0.12; 1.97	50.25 ± 0.11; 1.29
	55.0	54.91 ± 0.41; 0.72	54.92 ± 0.33; 2.11	55.36 ± 0.43; 1.88	55.31 ± 0.31; 0.98

Technique: UHPLC					
Vitamin D_3_	45.0	45.15 ± 0.12; 1.46	45.45 ± 0.22; 1.02	45.01 ± 0.02; 1.19	44.63 ± 0.21; 0.54
	50.0	50.12 ± 0.22; 0.80	50.33 ± 0.62; 0.79	50.33 ± 0.11; 0.42	49.41 ± 0.31; 0.39
	55.0	55.34 ± 0.32; 0.84	55.41 ± 0.32; 0.78	54.98 ± 0.21; 0.42	55.19 ± 0.12; 0.74
MK-7	45.0	45.21 ± 0.41; 1.20	45.23 ± 0.92; 1.67	45.04 ± 0.11; 1.64	45.23 ± 0.13; 2.21
	50.0	50.52 ± 0.51; 0.93	50.29 ± 0.11; 1.08	50.51 ± 0.21; 1.08	50.53 ± 0.21; 1.23
	55.0	55.31 ± 0.71; 0.97	55.61 ± 0.31; 0.54	55.29 ± 0.21; 0.81	55.31 ± 0.22; 1.35

SEM: standard error mean; RSD: relative standard deviation.

**Table 6:** Robustness study of vitamin D_3_ and MK-7 by UHPLC.

Chromatographic conditions	Vitamin D_3_				MK-7				
	Assay (%)	*t*_*R*_ (min)	*N*	TF	Assay (%)	*t*_*R*_ (min)	*N*	TF	Rs
Flow rate: 0.7	101.36	1.14	23540	0.95	101.23	3.34	28265	0.93	27.62
Flow rate: 0.5	101.29	1.37	23351	0.99	99.78	3.75	28233	0.98	27.59
(±0.1 mL min^−1^)									
Mobile phase (55 : 45)	100.17	1.26	23040	0.93	99.71	3.65	28865	0.99	27.62
Mobile phase (45 : 55)	99.53	1.25	23259	0.98	100.28	3.64	28223	0.93	27.61
(±5.0 mL)									
Column temp. (45°C)	99.39	1.22	23390	0.97	101.36	3.62	28444	0.96	27.62
Column temp. (35°C)	99.81	1.28	23287	0.96	100.51	3.61	28304	0.98	27.63
(±5°C)									
Wavelength (267 nm)	100.52	1.27	23289	0.99	100.32	3.65	28109	0.94	27.62
Wavelength (263 nm)	101.43	1.25	23401	0.96	99.83	3.63	28119	0.95	27.63
(±2 nm)									
pH: 6.1	99.87	1.27	23540	0.96	100.34	3.64	28165	0.94	27.62
pH: 5.9	100.18	1.25	23387	0.93	100.39	3.63	28338	0.95	27.65
(±0.1)									

*t*_*R*_: retention time, *N*: theoretical plates, TF: tailing factor, and Rs: resolution.

**Table 7:** Assay result of vitamin D_3_ and MK-7 by UHPLC in commercial tablet formulation.

Product	Contents	Label claim (*μ*g)	^a^Concentration found *μ*g ± SEM; RSD	Recovery (%)
Avelia	Vitamin D_3_	10	10.34 ± 0.12; 1.20	103.59
	MK-7	90	92.58 ± 0.11; 1.12	102.87

^a^Results are expressed as average of ten measurements.
